# Supplementary material for: Vitamin D Modified DSS-Induced Colitis in Mice via STING Signaling Pathway
Source: Biology (Basel). 2025 Jun 18;14(6):715. doi: 10.3390/biology14060715 (PMC12190092; doi:10.3390/biology14060715)
Supplement: Supplementary file 1 [file biology-14-00715-s001.zip › Supplementary Materials S1: 16s-report of gut microbiota/src/doc/16sV4method-en.pdf]

MATERIALS AND METHODS

Sample collection and satorage

As we known, different sample collection, storage methods even proposed sampling site will have an impact on the results of 16s sequencing of intestinal flora. Though there is conflicting evidence on whether different storage conditions alone can have an impact on microbial community studies (McKain et al., 2013, Lauber et al., 2011). It is often not practical to extract DNA from fresh samples; therefore, samples are generally stored for various flora stabilizing agent prior to DNA extraction. Consequently, when considering the use of a cryoprotectant for storage, it is important to ensure that all samples are stored in the same manner. The present study explored the effects of using GUHE Flora Storage buffer(Zhejiang Hangzhou Equipment Preparation 20190682, GUHE Laboratories, Hangzhou, China).

DNA Extraction

Total bacterial genomic DNA samples were extracted using the Metagenomic DNA was extracted from all Samples using the GHFDE100 (Zhejiang Hangzhou Equipment Preparation 20190952) DNA isolation kit (GUHE Laboratories, Hangzhou, China), following the manufacturer's instructions. Incidentally, This extraction method has also obtained a Chinese national invention patent (ZL201511009389.7). The quantity and quality of extracted DNAs were measured using a NanoDrop ND-1000 spectrophotometer (Thermo Fisher Scientific, Waltham, MA,USA) and agarose gel electrophoresis, respectively.

16S rDNA Amplicon Pyrosequencing

PCR amplification of the bacterial 16S rRNA genes V4 region was performed using the forward primer 515F (5'- GTGCCAGCMGCCGCGGTAA -3') and the reverse primer 806R (5'-GGACTACHVGGGTWTCTAAT-3'). Sample-specific paired-end 6-bp barcodes were incorporated into the TrueSeq adaptors for multiplex sequencing. The PCR components contained 25 μl of Phusion High-Fidelity PCR Master Mix, 3 μl (10 uM) of each Forward and Reverse primer, 10 μl of DNA Template, 3μl of DMSO, and 6 μl of ddH2O. Thermal cycling consisted of initial denaturation at 98 °C for 30 s, followed by 25 cycles consisting of denaturation at 98 °C for 15 s, annealing at 58 °C for 15 s, and extension at 72 °C for 15 s, with a final extension of 1 min at 72 °C. PCR amplicons were purified with Agencourt AMPure XP Beads (Beckman Coulter, Indianapolis, IN) and quantified using the PicoGreen dsDNA Assay Kit (Invitrogen, Carlsbad, CA, USA). After the individual quantification step, amplicons were pooled in equal amounts, and pair-end 2×150 bp sequencing was performed using the Illlumina NovaSeq6000 platform at GUHE Info technology Co., Ltd (Hangzhou,China).

Sequence Analysis

Raw sequencing reads with exact matches to the barcodes were assigned to respective samples and identified as valid sequences. Paired-end reads were assembled using Vsearch V2.22.1(--fastq_mergepairs --fastq_minovlen 5).The Amplicon Sequence Variant (ASV) picking using Vsearch v2.22.1, included dereplication (--derep_fulllength), Perform quality control and denoising sequences with UNOISE2 algorithm (--cluster_unoise) (Edgar, 2016) , Chimera removal (----uchime3_denovo) (Rognes 2016) and mapping to ASVs with 100% similarity threshold (--usearch_global).

To minimize the difference of sequencing depth across samples, normalization was performed on the ASV sequence counts of samples in the ASV Table, in order to ensure that the sum of ASV sequences in each sample was consistent. Normalized values were set to 1, representing relative abundances. A representative sequence (rep-seqs) was selected from each ASV using default parameters. Importing rep-seqs and ASV table files into QIIME2(QIIME2-2022.2) (Bolyen et al., 2019).ASVs containing less than 0.001% of total sequences across all samples were discarded by QIIME2. Taxonomic identifications represented the respective sequences assigned to ASVs against the QIIME2 weighted taxonomic classifiers (silva-138-99-nb-weighted-classifier). The taxonomy generated was collapsed (Levels 1–7) using the "qiime taxa collapse" command.

Bioinformatics and Statistical Analysis

Alpha and beta diversity calculations were generated using the q2-diversity core-metrics phylogenetic tool from a ASV table. Alpha-diversity metrics included Chao1 richness estimator, ACE metric (Abundance-based Coverage Estimator), PD_whole_tree ,Shannon diversity index and Simpson index, with statistically significant differences between groups identified using the Kruskal–Wallis test. For beta diversity, weighted UniFrac distances ,unweighted UniFrac distances(Lozupone and Knight 2005, Lozupone, Hamady et al. 2007) and Bray-Curtis distance was used for ordination via principal coordinates analysis (PCoA), with statistically significant differences between groups identified using analysis of similarity (ANOSIM) and multivariate variance analysis (Adonis) also called Permutational multivariate analysis of variance (PERMANOVA) (McArdle and Anderson 2001) using R package "vegan". Beta diversity analysis visualized via principal coordinate analysis (PCoA),principal component analysis (PCA) and nonmetric multidimensional scaling (NMDS) (Ramette 2007).

Sequence data analyses were mainly performed using QIIME2 and R packages (v3.6.3). Venn diagram was generated to visualize the shared and unique ASVs among samples or groups using R package "VennDiagram", based on the occurrence of ASVs across samples/groups regardless of their relative abundance (Zaura, Keijser *et al*. 2009).Principal component analysis (PCA) was also conducted based on the ASV-level compositional profiles (Ramette 2007). The envfit function in the VEGAN R package was used to perform a permutation test to test for effects of environmental factors on community structure.

Taxa abundances at the phylum, class, order, family, genus and species levels were statistically compared among groups by Kruskal–Wallis rank-sum test (groups = 2) or Wilcoxon rank-sum test (groups > 2) from R stats package.Taxa abundances were tested for between-group differences using the Kruskal-Wallis test or Tukey test, and visualized through the box-and-whiskers plots. And taxa abundances was compared with the sample groups in pairs with the MetagenomeSeq method (R metagenomeSeq package) (Paulson et al., 2013). All statistical tests P < 0.05 was considered statistically significant.

根据分组情况选择
对应的统计方法描述

报告中有3种统计检验方法，
MetagenomeSeq最严格
KS检验最常规
Trukey要求各组样本数量一致
文中可选择其中一种统计方法
便于方法统一和结果的可比较

LEfSe (Linear discriminant analysis effect size) was performed to detect differentially abundant taxa across groups using the default parameters (Segata, Izard *et al*. 2011). Random forest analysis was applied to discriminating the samples from different groups using the R package "randomForest" with 1,000 trees and all default settings (Breiman 2001, Liaw and Wiener 2002). The generalization error was estimated using 10-fold cross-validation. The expected "baseline" error was also included, which was obtained by a classifier that simply predicts the most common category label. Analysis of receiver operation characteristics (ROCs) and areas under the curve (AUCs) were used to assess the accuracy of the diagnostic model. Function prediction analysis(KEGG enzymes and MetaCyc pathway) was performed using PICRUSt2 (Douglas et al., 2020). Similarly, FAPROTAX and BugBase was used to predict function from taxonomic profiles.The output file was further analysed using Statistical Analysis of Metagenomic Profiles (STAMP) software package v2.1.3(Parks *et al*., 2014).

Data Access

All raw sequences were deposited in the NCBI Sequence Read Archive under accession number SRP******.

References

Breiman, L. (2001). "Random forests." Machine Learning 45(1): 5-32.

Bolyen, E., Rideout, J. R., Dillon, M. R., Bokulich, N. A., Abnet, C. C., Al-Ghalith, G. A., Alexander, H., Alm, E. J., Arumugam, M., Asnicar, F., et al. (2019). Reproducible, interactive, scalable and extensible microbiome data science using QIIME 2. Nat Biotechnol 37(8): 852-857.

Chen, H. and W. Jiang (2014). "Application of high-throughput sequencing in understanding human oral microbiome related with health and disease." Frontiers in Microbiology 5: 6.

Chen, Y. F., F. L. Yang, H. F. Lu, B. H. Wang, Y. B. Chen, D. J. Lei, Y. Z. Wang, B. L. Zhu and L. J. Li (2011).”Characterization of Fecal Microbial Communities in Patients with Liver Cirrhosis.” Hepatology 54(2):562-572.

Fadrosh, D.W., Ma, B., Gajer, P., Sengamalay, N., Ott, S., Brotman, R.M. and Ravel. J. (2014) “An improved dual-indexing approach for multiplexed 16S rRNA gene sequencing on the Illumina MiSeq platform. “ Microbiome 2:6.

Gill, S. R., M. Pop, R. T. DeBoy, P. B. Eckburg, P. J. Turnbaugh, B. S. Samuel, J. I. Gordon, D. A. Relman, C. M. Fraser-Liggett and K. E. Nelson (2006). “Metagenomic analysis of the human distal gut microbiome.” Science 312(5778): 1355-1359.

Douglas G. M., Maffei V. J., Zaneveld J. R., Yurgel S. N., Brown J. R., Taylor C. M., et al. . (2020). PICRUSt2 for prediction of metagenome functions. Nat. Biotechnol. 38, 685–688.

Liaw, A. and M. Wiener (2002). “Classification and regression by randomForest.” R News 2(3): 18-22.

Lozupone, C. and R. Knight (2005). “UniFrac: a new phylogenetic method for comparing microbial communities.” Applied and environmental microbiology 71(12): 8228-8235.

Lozupone, C. A., M. Hamady, S. T. Kelley and R. Knight (2007). “Quantitative and qualitative beta diversity measures lead to different insights into factors that structure microbial communities.” Applied and Environmental Microbiology 73(5): 1576-1585.

Parks, D.H., Tyson, G.W., Hugenholtz, P., Beiko, R.G. (2014). “STAMP: Statistical analysis of taxonomic and functional profiles.” Bioinformatics 30:3123-3124.

Bokulich N. A., Kaehler B. D., Rideout J. R., Dillon M., Bolyen E., Knight R., et al. (2018a). Optimizing Taxonomic Classification of Marker-Gene Amplicon Sequences with QIIME 2's Q2-Feature-Classifier Plugin. Microbiome 6, 470. 10.1186/s40168-018-0470-z.

McArdle, B. H. and M. J. Anderson (2001). “Fitting multivariate models to community data: a comment on distance-based redundancy analysis.” Ecology 82(1): 290-297.

Ramette, A. (2007). “Multivariate analyses in microbial ecology.” Fems Microbiology Ecology 62(2):142-160.

Edgar RC. (2016). “UNOISE2: improved error-correction for Illumina 16S and ITS amplicon sequencing. ” bioRxiv doi:10.1101/003723.

Rognes, T., Flouri, T., Nichols, B., Quince, C., Mahé, C (2016). “VSEARCH: a versatile open source tool for metagenomics. “PeerJ 4: e2584.

Paulson,J.N. et al. (2013) Differential abundance analysis for microbial marker-gene surveys. Nat. Methods, 10, 1200–1202.

Segata, N., J. Izard, L. Waldron, D. Gevers, L. Miropolsky, W. S. Garrett and C. Huttenhower (2011). “Metagenomic biomarker discovery and explanation.” Genome Biology 12(6).

McKain N, Genc B, Snelling TJ, Wallace RJ. 2013. Differential recovery of bacterial and archaeal 16S rRNA genes from ruminal digesta in response to glycerol as cryoprotectant. J Microbiol Methods 95:381–383.

Lauber CL, Zhou N, Gordon JI, Knight R. 2011. Effect of storage conditions on the assessment of bacterial community structure in soil and human-associated samples. FEMS Microbiol Lett 307:80–86.

Shannon, P., A. Markiel, O. Ozier, N. S. Baliga, J. T. Wang, D. Ramage, N. Amin, B. Schwikowski and T. Ideker (2003). “Cytoscape: A software environment for integrated models of biomolecular interaction networks.” Genome Research 13(11): 2498-2504.

Warton, D. I., S. T. Wright and Y. Wang (2012). “Distance-based multivariate analyses confound location and dispersion effects.” Methods in Ecology and Evolution 3(1): 89-101.

Zaura, E., B. J. F. Keijser, S. M. Huse and W. Crielaard (2009). “Defining the healthy “core microbiome” of oral microbial communities.” Bmc Microbiology 9: 12.
